# Multidimensional assessment of patient-reported outcomes and TCM syndrome improvement in allergic rhinitis: A retrospective cohort study of acupuncture combined with Yu Ping Feng San

**DOI:** 10.1097/MD.0000000000044429

**Published:** 2025-10-10

**Authors:** Jingquan Wang, Zhifang Wang

**Affiliations:** a Department of Rehabilitation Medicine, The Affiliated Changsha Central Hospital, Hengyang Medical School, University of South China, Changsha, Hunan, China; b Department of Integrated TCM and Western Medicine, The Affiliated Changsha Central Hospital, Hengyang Medical School, University of South China, Changsha, Hunan, China.

**Keywords:** acupuncture, allergic rhinitis, traditional Chinese medicine syndrome, Yu Ping Feng San

## Abstract

Allergic rhinitis (AR) is a common chronic condition that significantly impairs quality of life. Traditional Chinese medicine (TCM) recognizes lung qi deficiency and cold-type syndrome as a prevalent subtype. This study aimed to provide real-world evidence on the efficacy and safety of acupuncture combined with Yu Ping Feng San in treating this TCM subtype. A retrospective cohort study was conducted involving 91 patients with lung qi deficiency and cold-type AR treated at the Central Hospital of Changsha between January 2024 and May 2025. Patients were divided into an observation group (acupuncture + Yu Ping Feng San, n = 45) and a control group (fluticasone propionate nasal spray, n = 46). Primary outcomes included total efficacy rate, total nasal symptom score (TNSS), rhinoconjunctivitis quality of life questionnaire (RQLQ) score, TCM syndrome score, and serum total IgE level, evaluated at baseline, after 4 weeks of treatment, and at 4 weeks posttreatment follow-up. Adverse events were also recorded. After 4 weeks, the observation group showed a significantly higher total efficacy rate (84.44%) compared to the control group (65.22%) (χ² = 4.452, *P* = .035). TNSS and RQLQ scores were significantly lower in the observation group (4.29 ± 1.38 and 43.92 ± 14.33) versus the control group (5.18 ± 1.43, *P* = .003; 52.73 ± 13.47, *P* = .003). At follow-up, TNSS and RQLQ scores slightly increased but remained significantly better in the observation group (*P* < .001). Posttreatment serum IgE levels were also significantly lower in the observation group (113.51 ± 28.57 IU/mL vs 134.82 ± 30.73 IU/mL, *P* = .001). Adverse event rates were comparable (13.3% vs 15.2%, *P* = .797), with no serious events reported. Acupuncture combined with Yu Ping Feng San demonstrates superior efficacy in relieving symptoms, improving quality of life, and reducing serum IgE levels in lung qi deficiency and cold-type AR compared to fluticasone propionate alone. The therapy is well tolerated and shows sustained benefits posttreatment.

## 1. Introduction

Allergic rhinitis (AR) is a chronic inflammatory disease mediated by immunoglobulin E (IgE), characterized primarily by nasal congestion, rhinorrhea, sneezing, and nasal itching. These symptoms significantly impair patients’ quality of life and may also lead to concentration difficulties, sleep disturbances, and mood fluctuations.^[1–3]^ Epidemiological data show that the prevalence of AR ranges from approximately 11.9% to 30.2% in Western countries, while the prevalence of persistent AR among Chinese adults is also as high as 26%.^[4–6]^ The chronic and recurrent nature of AR not only increases the economic burden on patients and their families but also places substantial pressure on healthcare systems. It has been reported that annual drug-related expenditures for AR account for nearly 22% of patients’ income.^[7]^ Therefore, achieving effective, long-lasting, and safe symptom control remains a pressing challenge in clinical practice.

Current Western medical management of AR mainly includes antihistamines, topical or systemic corticosteroids, mast cell stabilizers, leukotriene receptor antagonists, and allergen-specific immunotherapy.^[8–10]^ Although these approaches can provide significant short-term relief of nasal symptoms, long-term or combined use is often associated with adverse effects such as dry mouth, drowsiness, local irritation, abnormal vascular reactivity, and decreased bone density. These issues contribute to reduced patient adherence and hinder sustained efficacy.^[11–14]^ Furthermore, Western medicine lacks a constitution-based approach to regulate overall immune function, and is often less effective in individuals with lung qi deficiency and cold syndrome. As a result, conventional pharmacotherapy alone may not adequately meet patients’ needs for long-term safety and holistic regulation.

According to the theory of traditional Chinese medicine (TCM), AR falls under the category of “Bi Qiu” (nasal congestion and discharge) and is primarily attributed to dysfunction of the lung, spleen, and kidney, with lung qi deficiency and cold being the most common syndrome pattern.^[15–17]^ The combined use of acupuncture and Chinese herbal formulations has demonstrated unique advantages in harmonizing qi and blood as well as strengthening the body’s defenses. Yu Ping Feng San is traditionally employed to tonify qi, consolidate the exterior, and regulate defensive qi; acupuncture, by stimulating meridians, can improve local blood flow, restore nasal mucosal function, and activate systemic immune responses, thereby achieving holistic regulation.^[18–20]^ In recent years, multiple studies and clinical guidelines have recommended acupuncture combined with Yu Ping Feng San or similar prescriptions for the treatment of AR. However, most of these studies lack systematic retrospective evaluations based on TCM syndrome differentiation.^[21–23]^

Therefore, this study aimed to evaluate the clinical efficacy and safety of acupuncture combined with Yu Ping Feng San in patients with lung qi deficiency and cold-type AR, compared to conventional intranasal corticosteroid therapy. We hypothesized that the combined therapy would lead to superior improvement in clinical symptoms, TCM syndrome scores, quality of life, and immunological markers (serum IgE), with sustained benefits during follow-up. The core variables assessed included treatment modality (intervention vs control), total nasal symptom score (TNSS), rhinoconjunctivitis quality of life questionnaire (RQLQ) score, TCM syndrome score, and serum total IgE level. Through a multidimensional outcome evaluation, this study sought to provide empirical evidence supporting syndrome-based integrative approaches to AR management.

## 2. Materials and methods

### 2.1. Study design

This study adopted a retrospective cohort design to evaluate the clinical efficacy of a TCM-based regimen involving acupuncture combined with Yu Ping Feng San in patients with lung qi deficiency and cold-type AR. The study was approved by the institutional ethics committee and adhered strictly to relevant privacy protection protocols. No direct patient intervention was involved; all data were obtained through review and analysis of electronic medical records and treatment documentation. All collected data were anonymized and de-identified, and used solely for academic publication purposes.

### 2.2. Diagnostic criteria

TCM diagnosis was based on the *Guidelines for the Diagnosis and Treatment of Common Diseases in TCM Otorhinolaryngology,*^[24]^ incorporating typical manifestations of lung qi deficiency and cold-type AR, such as nasal itching, frequent sneezing, profuse clear nasal discharge, and nasal congestion. These were supplemented with tongue features (pale tongue with white coating), weak pulse, spontaneous sweating, and aversion to cold, while excluding other common nasal conditions. The Western medicine diagnosis followed the *Guidelines for the Diagnosis and Treatment of AR (2022 Revision),*^[25]^ and was based on clinical features (e.g., sneezing, watery rhinorrhea, nasal itching, and nasal congestion) and auxiliary examinations (e.g., serum-specific IgE or skin prick tests), with exclusion of other types of rhinitis or related disorders.

### 2.3. Study population

Eligible patients were those diagnosed between January 2024 and May 2025 in the Departments of Integrated Traditional Chinese and Western Medicine, Otorhinolaryngology–Head and Neck Surgery, Rehabilitation Medicine, and Pulmonology at the Central Hospital of Changsha, affiliated with the University of South China. Relevant clinical data were retrieved retrospectively from outpatient/emergency and inpatient medical records, diagnostic notes, laboratory results, and follow-up documentation. Based on the received treatment, patients were assigned to 2 groups: the observation group received acupuncture combined with Yu Ping Feng San, while the control group was treated with intranasal corticosteroid fluticasone propionate spray. According to inclusion and exclusion criteria, a total of 91 patients were enrolled, with 45 in the observation group and 46 in the control group.

#### 2.3.1. Inclusion criteria

Patients aged 18 to 65 years with a documented diagnosis of AR and TCM syndrome differentiation as lung qi deficiency and cold-type. TNSS ≥ 7 at initial diagnosis. All included patients had undergone acupuncture combined with Yu Ping Feng San, had complete medical records with clear efficacy documentation, met both TCM and Western diagnostic criteria, and complied with ethical and privacy standards.

#### 2.3.2. Exclusion criteria

Patients with comorbid persistent asthma, chronic obstructive pulmonary disease, or active malignancy; history of asthma-related emergency visits or hospitalizations within the past 12 months; previous allergen-specific immunotherapy or anti-IgE monoclonal antibody use; coexisting acute upper respiratory tract infections, sinusitis, or other infections during treatment; known allergies to acupuncture, components of Yu Ping Feng San, or fluticasone; or incomplete records that prevented efficacy evaluation.

### 2.4. Treatment protocols

Patients in the observation group received acupuncture combined with Yu Ping Feng San. Acupoints included Dazhui (GV14), Yintang (EX-HN3), Sibai (ST2), Yingxiang (LI20), Shangyingxiang (EX-HN8), Shangxing (GV23), Chize (LU5, bilateral), and Hegu (LI4, bilateral), selected according to the national standard *Nomenclature and Location of Acupoints* (GB/T 12346-2006).^[26]^ Acupuncture was performed once daily, 5 sessions per week with 2 days of rest, for a total treatment duration of 4 weeks. The herbal formula, a modified Yu Ping Feng San, included Fangfeng, Huangqi, Baizhu, Cang’erzi, Xinyi, Baizhi, Wuweizi, Xixin, and Jiegeng. The decoction was administered once daily, divided into morning and evening doses, for 4 weeks.

Control group patients were treated with fluticasone propionate nasal spray (50 μg/spray, manufactured by GlaxoSmithKline), administered as 2 sprays per nostril daily (total 200 μg/day) for 4 weeks. This regimen aligns with domestic and international AR treatment guidelines, which recommend intranasal corticosteroids as first-line therapy. The treatment duration and follow-up period matched those of the observation group.

#### 2.4.1. Supplementary note

In the retrospective analysis, control group patients had no records of acupuncture or herbal treatments. Comorbid chronic diseases and related medications were documented in their records. Some control group patients with poor symptom control received additional antihistamines, with dosage and frequency recorded during data collection.

### 2.5. Outcome measures

Treatment efficacy was assessed at baseline, end of treatment, and end of follow-up using the following 6 indicators:

1. Overall therapeutic efficacy: Based on the *Guidelines for the Diagnosis and Treatment of AR (2022 Revision).*^[25]^ Markedly effective: efficacy index ≥ 66%; effective: 26% to 65%; ineffective: ≤25%. Total efficacy rate = (markedly effective + effective)/ total cases × 100%.
2. TCM syndrome score: Evaluated using a TCM symptom scoring scale with 9 items rated from 0 to 3, total score 0 to 27. Higher scores indicate more severe symptoms, reflecting changes in lung qi deficiency and cold syndrome.
3. TNSS: TNSS assessed the severity of sneezing, rhinorrhea, nasal congestion, and nasal itching. Total score ranges from 0 to 12, with higher scores indicating more severe symptoms and reflecting clinical improvement in AR.
4. RQLQ: RQLQ was used to assess changes in patient quality of life before and after treatment. It contains 28 items across 7 domains: most troublesome symptoms, sleep, nonnasal/eye symptoms, practical problems, nasal symptoms, eye symptoms, and emotional function. Each item is rated 0 to 6, for a total score range of 0 to 168; higher scores reflect greater quality of life impairment.
5. Serum-specific IgE detection: Specific IgE levels for relevant allergens were used to assess allergic status. Levels ≥ 0.35 kU/L were considered positive and were used to assist diagnosis and evaluate therapeutic response.
6. Adverse event rate: Adverse events during treatment were recorded, including subcutaneous bruising, gastrointestinal discomfort, epistaxis, dry mouth, dizziness, etc, to assess treatment safety.

### 2.6. Statistical analysis

All data were analyzed using SPSS version 26.0. Continuous variables were expressed as mean ± standard deviation (X ± SD), and categorical variables as frequency (n) and percentage (%). For continuous variables, *t*-tests were applied based on distribution characteristics. Categorical variables were compared using the chi-square test. *P* < .05 was considered statistically significant.

## 3. Results

### 3.1. General characteristics

There were no statistically significant differences in baseline characteristics between the observation and control groups, indicating good comparability. The mean age was 35.92 ± 6.47 years in the observation group and 37.78 ± 7.43 years in the control group (*t* = –1.272, *P* = .207). The average disease duration was 5.93 ± 3.18 years and 5.46 ± 2.47 years, respectively (*t* = 0.788, *P* = .433). The distribution of AR severity showed no significant difference (mild: 46.67% vs 63.04%; moderate-to-severe: 53.33% vs 36.96%; χ² = 2.464, *P* = .116). The gender distribution was also similar between the groups (χ² = 0.537, *P* = .464) (Table 1).

**Table 1:** Comparison of baseline characteristics between the 2 groups (X ± SD, n/%).

Variable	Observation group (n = 45)	Control group (n = 46)	*t*/χ²	*P*
Age (years)	35.92 ± 6.47	37.78 ± 7.43	–1.272	.207
Gender				
Male	24 (53.33%)	21 (45.65%)	0.537	.464
Female	21 (46.67%)	25 (54.35%)		
Disease duration (year)	5.93 ± 3.18	5.46 ± 2.47	0.788	.433
AR severity				
Mild	21 (46.67%)	29 (63.04%)	2.464	.116
Moderate/severe	24 (53.33%)	17 (36.96%)		

AR = allergic rhinitis, SD = standard deviation.

### 3.2. Clinical efficacy

After 4 weeks of treatment, the total efficacy rate in the observation group was 84.44%, significantly higher than 65.22% in the control group (χ² = 4.452, *P* = .035), suggesting that acupuncture combined with Yu Ping Feng San was more effective in alleviating AR symptoms (Table 2).

**Table 2:** Comparison of clinical efficacy between the 2 groups (n/%).

Group	Markedly effective	Effective	Ineffective	Total efficacy rate
Observation group (n = 45)	6 (13.33%)	32 (71.11%)	7 (15.56%)	38 (84.44%)
Control group (n = 46)	3 (6.52%)	27 (58.70%)	16 (34.78%)	30 (65.22%)
χ²	–	–	–	4.452
*P*	–	–	–	.035

### 3.3. TCM syndrome score

Posttreatment, TCM syndrome scores decreased significantly in both groups compared to baseline (*P* < .05), indicating clinical improvement. The score in the observation group declined from 16.88 ± 2.23 to 7.25 ± 2.09, while in the control group, it decreased from 17.60 ± 2.18 to 11.13 ± 2.17. The greater reduction in the observation group was statistically significant (*t* = –8.689, *P* <.001). At the end of follow-up, the score in the observation group slightly increased to 9.50 ± 1.73 but remained significantly lower than 13.62 ± 1.92 in the control group (*t* = –10.747, *P* < .001). These results suggest that the combined therapy not only improved TCM syndrome scores more effectively but also maintained its benefits over time (Table 3).

**Table 3:** Comparison of TCM syndrome scores before and after treatment between the 2 groups (X ± SD).

Time point	Observation group (n = 45)	Control group (n = 46)	*t*	*P*
Before treatment	16.88 ± 2.23	17.60 ± 2.18	–1.557	.122
After treatment	7.25 ± 2.09^*^	11.13 ± 2.17^*^	–8.689	<.001
At follow-up	9.50 ± 1.73^#^	13.62 ± 1.92^#^	–10.747	<.001

SD = standard deviation, TCM = traditional Chinese medicine.

* Compared with values before treatment within the same group, *P* <.05.

** Compared with values after treatment within the same group, *P* <.05.

### 3.4. TNSS score

Before treatment, there was no significant difference in TNSS between the 2 groups (9.96 ± 1.28 vs 10.00 ± 2.14; *t* = –0.108, *P* = .914). After 4 weeks, TNSS scores in both groups significantly decreased from baseline, indicating symptom relief. The observation group showed a greater reduction (4.29 ± 1.38) than the control group (5.18 ± 1.43), with the difference being statistically significant (*t* = –3.021, *P* = .003). At the end of follow-up, the TNSS in the observation group slightly increased to 4.95 ± 1.92, whereas in the control group it increased more markedly to 6.61 ± 1.78, which was significantly higher than the observation group (*t* = –4.275, *P* < .001). These findings demonstrate that the combined treatment provided more substantial and longer-lasting symptom relief (Table 4).

**Table 4:** Comparison of TNSS scores before and after treatment between the 2 groups (X ± SD).

Time point	Observation group (n = 45)	Control group (n = 46)	*t*	*P*
Before treatment	9.96 ± 1.28	10.00 ± 2.14	–0.108	.914
After treatment	4.29 ± 1.38*	5.18 ± 1.43*	–3.021	.003
At follow-up	4.95 ± 1.92	6.61 ± 1.78**	–4.275	<.001

SD = standard deviation, TNSS = total nasal symptom score.

* Compared with values before treatment within the same group, *P* <.05.

** Compared with values after treatment within the same group, *P* <.05.

### 3.5. RQLQ score

Pretreatment RQLQ scores were comparable between the 2 groups (75.90 ± 15.32 vs 73.46 ± 19.27; *t* = 0.669, *P* = .504), indicating similar baseline quality of life related to rhinoconjunctivitis. After 4 weeks of treatment, both groups experienced marked improvements. The observation group achieved a greater reduction in RQLQ score (43.92 ± 14.33) than the control group (52.73 ± 13.47), with statistical significance (*t* = –3.022, *P* = .003). At 4 weeks posttreatment, the score in the observation group slightly rebounded to 47.98 ± 11.22 but remained significantly lower than 57.80 ± 10.76 in the control group (*t* = –4.259, *P* < .001). This indicates that acupuncture combined with Yu Ping Feng San not only improved quality of life during treatment but also sustained its benefits posttreatment (Table 5).

**Table 5:** Comparison of RQLQ scores between groups at different time points (X ± SD).

Time point	Observation group (n = 45)	Control group (n = 46)	*t*	*P*
Before treatment	75.90 ± 15.32	73.46 ± 19.27	0.669	.504
After treatment	43.92 ± 14.33^*^	52.73 ± 13.47*	–3.022	.003
At follow-up	47.98 ± 11.22	57.80 ± 10.76	–4.259	<.001

RQLQ = rhinoconjunctivitis quality of life questionnaire, SD = standard deviation.

* Compared with values before treatment within the same group, *P* <.05.

### 3.6. Serum IgE level

At baseline, there was no significant difference in total serum IgE between the 2 groups (237.83 ± 35.71 vs 232.63 ± 30.91 IU/mL; *t* = 0.742, *P* = .462). After treatment, IgE levels in the observation group decreased to 113.51 ± 28.57 IU/mL, which was significantly lower than 134.82 ± 30.73 IU/mL in the control group (*t* = –3.429, *P* = .001), suggesting a more pronounced immunoregulatory effect of the combined therapy (Table 6).

**Table 6:** Comparison of serum total IgE levels between the 2 groups after treatment (X ± SD, kU/L).

Time point	Observation group (n = 45)	Control group (n = 46)	*t*	*P*
Before treatment	237.83 ± 35.71	232.63 ± 30.91	0.742	.462
After treatment	113.51 ± 28.57^*^	134.82 ± 30.73*	–3.429	.001

IgE = immunoglobulin E, SD = standard deviation.

* Compared with values before treatment within the same group, *P* <.05.

### 3.7. Incidence of adverse events

The total incidence of adverse events was 13.33% in the observation group and 15.22% in the control group, with no significant difference (χ² = 0.066, *P* = .797). Common adverse events included subcutaneous hematoma, gastrointestinal discomfort, epistaxis, dry mouth, and dizziness. All were mild and well tolerated. No serious adverse events were reported (Table 7).

**Table 7:** Comparison of adverse reaction incidence between the 2 groups (n/%).

Group	Subcutaneous hematoma	Gastrointestinal discomfort	Epistaxis	Dry mouth	Dizziness	Total incidence of adverse reactions
Observation group (n = 45)	2 (4.44%)	2 (4.44%)	0 (0.00%)	1 (2.22%)	1 (2.22%)	6 (13.33%)
Control group (n = 46)	0 (0.00%)	2 (4.35%)	1 (2.17%)	3 (6.52%)	1 (2.17%)	7 (15.22%)
χ²	–	–	–	–	–	0.066
*P*	–	–	–	–	–	.797

## 4. Discussion

This study systematically evaluated the clinical application of acupuncture combined with Yu Ping Feng San in patients with lung qi deficiency and cold-type AR based on retrospective cohort data. The results demonstrated that the combined treatment was superior to fluticasone propionate nasal spray monotherapy in relieving nasal symptoms, improving quality of life, and modulating immune function, while maintaining a favorable safety profile.

The total efficacy rate in the observation group reached 84.44%, significantly higher than 65.22% in the control group. Multiple outcome indicators – including TCM syndrome score, TNSS, and RQLQ score – remained superior in the observation group at both posttreatment and follow-up stages, indicating rapid onset and stable efficacy. In addition, the serum total IgE level, an immunological marker, showed a significant reduction in the observation group, suggesting that the therapy may positively regulate allergen-specific immune responses. The incidence of adverse events was comparable between groups, and no serious adverse events were reported, further supporting the clinical feasibility and safety of this approach.

Importantly, this study not only verified the efficacy advantage of the combined intervention but also emphasized its foundation in TCM syndrome differentiation. Specifically, the identification of “lung qi deficiency and cold” as the core pathogenesis matched the therapeutic principle of tonifying qi, consolidating the exterior, and warming yang to unblock meridians.^[27,28]^ Unlike traditional drug interventions, acupuncture may act through holistic regulation of the meridian system, potentially influencing the neuroimmune network at the central level, enhancing visceral function, and improving the body’s regulation of allergic responses. Yu Ping Feng San, by addressing the deficiency of vital qi, strengthens the exterior and enhances systemic immune tolerance.^[29,30]^ Together, these therapies may address both “acute symptom control” and “long-term constitution regulation,” a dual-target strategy that has not been sufficiently emphasized in previous research.^[31]^

The retrospective cohort design of this study reflects real-world clinical settings. Data were derived from multidisciplinary outpatient and inpatient cases, and outcome measures included symptom scales, physiological immune markers, and quality of life scores, with posttreatment follow-up included to enhance the temporal understanding of treatment efficacy. This design helps mitigate limitations seen in some clinical studies, such as overly narrow interventions, limited assessment dimensions, or highly selective populations that compromise generalizability despite strong efficacy.^[32]^

Several limitations should be acknowledged. First, this was a single-center retrospective study, which may have introduced selection bias and limited generalizability. Second, although efforts were made to include patients with complete and comparable records, certain confounding factors such as comorbid chronic diseases and differences in medication adherence may have affected clinical outcomes. For instance, some control group patients received additional antihistamines due to poor symptom control, which may have influenced TNSS and RQLQ scores. Third, the sample size was relatively small, potentially reducing the statistical power to detect subtle differences. Fourth, we did not include objective evaluation tools such as nasal endoscopy, mucosal imaging, or inflammatory cytokine profiling, which limited the depth of mechanistic analysis. Finally, as the study was conducted in a single hospital, findings may not be generalizable to other clinical settings with different population characteristics or treatment protocols. Future multicenter, prospective studies with larger sample sizes and enhanced methodological rigor are warranted to validate and extend our findings.

In summary, the findings suggest that acupuncture combined with Yu Ping Feng San offers significant clinical value in treating patients with lung qi deficiency and cold-type AR. Its holistic therapeutic effects are evident not only in symptom relief but also in potential long-term modulation of constitution and immune status. Future research should include large-sample, multicenter prospective studies to explore the underlying mechanisms in greater depth and perform stratified analyses based on individual differences, thereby advancing the standardization and precision of integrated TCM and Western medicine in AR treatment.

## Author contributions

**Conceptualization:** Jingquan Wang, Zhifang Wang.

**Data curation:** Jingquan Wang, Zhifang Wang.

**Formal analysis:** Zhifang Wang.

**Funding acquisition:** Jingquan Wang.

**Investigation:** Jingquan Wang, Zhifang Wang.

**Methodology:** Jingquan Wang, Zhifang Wang.

**Supervision:** Jingquan Wang, Zhifang Wang.

**Validation:** Jingquan Wang, Zhifang Wang.

**Visualization:** Jingquan Wang, Zhifang Wang.

**Writing** – **original draft:** Jingquan Wang, Zhifang Wang.

**Writing** – **review & editing:** Jingquan Wang, Zhifang Wang.

## References

[1] Zhang Y Lan F Zhang L. Advances and highlights in allergic rhinitis. Allergy. 2021;76:3383–9. 34379805 10.1111/all.15044

[2] Dykewicz MS Wallace DV Baroody F; Workgroup Chair and Cochair. Treatment of seasonal allergic rhinitis: an evidence-based focused 2017 guideline update. Ann Allergy Asthma Immunol. 2017;119:489–511.e41. 29103802 10.1016/j.anai.2017.08.012

[3] Liu J Zhang X Zhao Y Wang Y. The association between allergic rhinitis and sleep: a systematic review and meta-analysis of observational studies. PLoS One. 2020;15:e0228533. 32053609 10.1371/journal.pone.0228533 PMC7018032

[4] Bauchau V Durham SR. Prevalence and rate of diagnosis of allergic rhinitis in Europe. Eur Respir J. 2004;24:758–64. 15516669 10.1183/09031936.04.00013904

[5] Zhang L Han D Huang D. Prevalence of self-reported allergic rhinitis in eleven major cities in China. Int Arch Allergy Immunol. 2009;149:47–57. 19033732 10.1159/000176306

[6] Nathan RA Meltzer EO Derebery J. The prevalence of nasal symptoms attributed to allergies in the United States: findings from the burden of rhinitis in an America survey. Allergy Asthma Proc. 2008;29:600–8. 19173786 10.2500/aap.2008.29.3179

[7] Cheng L Chen J Fu Q. Chinese Society of Allergy guidelines for diagnosis and treatment of allergic rhinitis. Allergy Asthma Immunol Res. 2018;10:300–53. 29949830 10.4168/aair.2018.10.4.300 PMC6021586

[8] Bousquet J Khaltaev N Cruz AA; World Health Organization. Allergic rhinitis and its impact on asthma (ARIA) 2008 update. Allergy. 2008;63:8–160. 18331513 10.1111/j.1398-9995.2007.01620.x

[9] Seidman MD Gurgel RK Lin SY; Guideline Otolaryngology Development Group. AAO-HNSF. Clinical practice guideline: allergic rhinitis. Otolaryngol Head Neck Surg. 2015;152:S1–43. 10.1177/0194599814561600 25644617

[10] Jutel M Agache I Bonini S. International consensus on allergy immunotherapy. J Allergy Clin Immunol. 2015;136:556–68. 26162571 10.1016/j.jaci.2015.04.047

[11] Máspero JF Rosenblut A Finn A Lim J Wu W Philpot E. Safety and efficacy of fluticasone furoate in pediatric patients with perennial allergic rhinitis. Otolaryngol Head Neck Surg. 2008;138:30–7. 18164990 10.1016/j.otohns.2007.10.023

[12] Meltzer EO Tripathy I Máspero JF Wu W Philpot E. Safety and tolerability of fluticasone furoate nasal spray once daily in paediatric patients aged 6–11 years with allergic rhinitis: subanalysis of three randomized, double-blind, placebo-controlled, multicentre studies. Clin Drug Investig. 2009;29:79–86. 10.2165/0044011-200929020-00002 19133703

[13] Rosenblut A Bardin PG Muller B. Long-term safety of fluticasone furoate nasal spray in adults and adolescents with perennial allergic rhinitis. Allergy. 2007;62:1071–7. 17686110 10.1111/j.1398-9995.2007.01521.x

[14] Lanier B Kai G Marple B Wall GM. Pathophysiology and progression of nasal septal perforation. Ann Allergy Asthma Immunol. 2007;99:473–9; quiz 480. 18219827 10.1016/S1081-1206(10)60373-0

[15] Ma C Li SL Hao RB. Mechanisms and research progress of traditional Chinese medicine in treating allergic rhinitis. Chin J Basic Tradit Chin Med. 2019;25:1473–6.

[16] Hu H Ji Z Qiang X. Chinese medical injections for acute exacerbation of chronic obstructive pulmonary disease: a network meta-analysis. Int J Chron Obstruct Pulmon Dis. 2021;16:3363–86. 34949918 10.2147/COPD.S335579 PMC8691136

[17] Huang HY Zhang QX Zhong J. Distribution patterns of eight TCM syndromes in 1,216 pediatric allergic rhinitis cases in the Chengdu region. Shizhen J Tradit Chin Med. 2019;30:2515–8.

[18] Li YW Sun MQ. Effects of Yupingfeng San combined with Du-meridian moxibustion on serum IL-6, cAMP, and cGMP in patients with allergic rhinitis. Chin J Exp Formulas. 2018;24:163–7.

[19] Xu Y Zhang L Chen C. Investigation of the efficacy and potential pharmacological mechanism of Yupingfeng in treating chronic obstructive pulmonary disease: a meta-analysis and in silico study. J Ethnopharmacol. 2025;343:119441. 39914688 10.1016/j.jep.2025.119441

[20] Huang JZ Lin S. Oral Yupingfeng San combined with yongquan-point application for treating pediatric allergic rhinitis of lung-qi deficiency type: clinical observation of 35 cases. J Pediatr Tradit Chin Med. 2017;13:80–5.

[21] He M Qin W Qin Z Zhao C. Acupuncture for allergic rhinitis: a systematic review and meta-analysis. Eur J Med Res. 2022;27:58. 35462555 10.1186/s40001-022-00682-3 PMC9036742

[22] Chen S Guo SN Marmori F. Clinical practice guideline for allergic rhinitis treatment with acupuncture. Chin J Integr Med. 2021;27:83–90. 32170521 10.1007/s11655-020-3161-0

[23] Feng S Han M Fan Y. Acupuncture for the treatment of allergic rhinitis: a systematic review and meta-analysis. Am J Rhinol Allergy. 2015;29:57–62. 25590322 10.2500/ajra.2015.29.4116

[24] Chinese Society of Traditional Chinese Medicine. ZYYXH/T307-321-2012 guidelines for diagnosis and treatment of common diseases in TCM otorhinolaryngology. 2012.

[25] Li HL Ruan HY Zhang JZ. Qidi Buxu Fang combined with medicated-thread point moxibustion for treating 52 cases of lung-qi deficiency type allergic rhinitis. Global Tradit Chin Med. 2023;16:1668–71.

[26] Hu H Luo J Ma J. Efficacy and safety of *Sanfeng TongqiaoDiwan* in patients with allergic rhinitis: a single-arm clinical trial in China. Ann Transl Med. 2022;10:684. 35845528 10.21037/atm-22-2768 PMC9279765

[27] Min C Peng C Wei G. Moxibustion with Chinese herbal has good effect on allergic rhinitis. Int J Clin Exp Med. 2015;8:16480–7. 26629174 PMC4659062

[28] Wang Z Cai X Pang Z. Yupingfeng pulvis regulates the balance of T cell subsets in asthma mice. Evid Based Complement Alternat Med. 2016;2016:6916353. 27143988 10.1155/2016/6916353 PMC4842077

[29] Li N Guo Y Gong Y. The anti-inflammatory actions and mechanisms of acupuncture from acupoint to target organs via neuro-immune regulation. J Inflamm Res. 2021;14:7191–224. 34992414 10.2147/JIR.S341581 PMC8710088

[30] Nie J Jiang X Wang G. Yu-Ping-Feng-San alleviates inflammation in atopic dermatitis mice by TLR4/MyD88/NF-κB pathway. J Ethnopharmacol. 2024;329:118092. 38604509 10.1016/j.jep.2024.118092

[31] Xue CC Li CG Hügel HM Story DF. Does acupuncture or Chinese herbal medicine have a role in the treatment of allergic rhinitis? Curr Opin Allergy Clin Immunol. 2006;6:175–9. 16670510 10.1097/01.all.0000225156.29780.36

[32] Vogelberg C Klimek L Brüggenjürgen B Jutel M. Real-world evidence for the long-term effect of allergen immunotherapy: current status on database-derived European studies. Allergy. 2022;77:3584–92. 36074052 10.1111/all.15506 PMC10087412

